# Co-Incidence of Epstein–Barr Virus and High-Risk Human Papillomaviruses in Cervical Cancer of Syrian Women

**DOI:** 10.3389/fonc.2018.00250

**Published:** 2018-07-02

**Authors:** Hamda Al-Thawadi, Lina Ghabreau, Tahar Aboulkassim, Amber Yasmeen, Semir Vranic, Gerald Batist, Ala-Eddin Al Moustafa

**Affiliations:** ^1^College of Medicine, Qatar University, Doha, Qatar; ^2^Faculty of Medicine, Pathology Department, University of Aleppo, Aleppo, Syria; ^3^Syrian Research Cancer Centre of the Syrian Society against Cancer, Aleppo, Syria; ^4^Segal Cancer Centre, Lady Davis Institute for Medical Research of the Sir Mortimer B. Davis-Jewish General Hospital, Montreal, QC, Canada; ^5^Oncology Department, McGill University, Montreal, QC, Canada; ^6^College of Medicine and Biomedical Research Centre of Qatar University, Doha, Qatar

**Keywords:** Epstein–Barr virus, high-risk human papillomaviruses, cervical cancer, Syrian women, cancer phenotype

## Abstract

Epstein–Barr virus (EBV) has been recently shown to be co-present with high-risk human papillomaviruses (HPVs) in human cervical cancer; thus, these oncoviruses play an important role in the initiation and/or progression of this cancer. Accordingly, our group has recently viewed the presence and genotyping distribution of high-risk HPVs in cervical cancer in Syrian women; our data pointed out that HPVs are present in 42/44 samples (95%). Herein, we aim to explore the co-prevalence of EBV and high-risk HPVs in 44 cervical cancer tissues from Syrian women using polymerase chain reaction, immunohistochemistry, and tissue microarray analyses. We found that EBV and high-risk HPVs are co-present in 15/44 (34%) of the samples. However, none of the samples was exclusively EBV-positive. Additionally, we report that the co-expression of LMP1 and E6 genes of EBV and high-risk HPVs, respectively, is associated with poorly differentiated squamous cell carcinomas phenotype; this is accompanied by a strong and diffuse overexpression of Id-1 (93% positivity), which is an important regulator of cell invasion and metastasis. These data imply that EBV and HPVs are co-present in cervical cancer samples in the Middle East area including Syria and their co-presence is associated with a more aggressive cancer phenotype. Future investigations are needed to elucidate the exact role of EBV and HPVs cooperation in cervical carcinogenesis.

## Introduction

Cervical cancer is the fourth most common malignancy among women worldwide with approximately 528,000 new cases and 266,000 deaths each year estimated by the World Health Organization. Notably, most cervical cancer deaths (87%) occur in the developing countries. Currently, it is well known that the majority of cancer deaths are the result of metastasis, either directly due to tumor involvement of critical organs or indirectly due to therapeutic resistance and the inability of available therapy to control tumor progression (1). On the other hand, it is estimated that approximately 20% of human cancers could be linked to oncoviruses infection including Epstein–Barr virus (EBV) and high-risk human papillomaviruses (HPVs) especially types 16, 18, and 33 (2–4). EBV is a human gammaherpesvirus that infects more than 90% of the human adult population. Acute infection with EBV can cause infectious mononucleosis, and its latent state can lead to several types of human B-cell lymphomas and carcinomas, especially nasopharyngeal (5, 6).

Today, it is well established that high-risk of HPVs infections are important etiological factors in the development of human cervical cancer; as more than 96% of cervical cancers are positive for high-risk HPVs especially types 16, 18, 31, 33, and 35 worldwide including the Middle East region (3, 7). Moreover, accumulating evidence suggests that persistent infection with these viruses is necessary for cervical precursors to evolve into invasive carcinomas (8). Accordingly, we have explored the presence of high-risk HPVs in cervical cancer in Syrian women; our study revealed that 95% of our samples are positive for HPVs; more significantly, we noted that the most frequent high-risk HPV types in Syrian women are 33, 16, 18, 45, 52, 58, and 35, in descending order. Furthermore, the expression of E6 onco-protein of high-risk HPVs was found to be correlated with the overexpression of Id-1, which is a member of the inhibitor of DNA-binding (Id) proteins (9).

Id proteins constitute a family of highly preserved transcriptional controllers that play critical roles during normal development and in the maintenance of homeostasis in human tissue (10). The main biological properties of Id proteins are inhibition of differentiation and conservation of the self-renewal capability and multipotency of stem cells (11). Id proteins are overexpressed in several human carcinomas (11, 12). More specifically, Id-1 protein expression is directly involved in cancer initiation and/or progression in different types of human malignancies including cervical (9, 13–15). On the other hand, it has been pointed out that LMP1 onco-protein of EBV upregulates Id-1 expression in nasopharyngeal immortalized and cancer cells (16, 17); however, the association between EBV onco-proteins and Id-1 in human carcinomas, including cervical is not clear.

Earlier studies have indicated that EBV is frequently present in human cervical cancer tissues, suggesting EBV is associated with the development of cervical cancer (18). Moreover, it has been shown that the co-occurrence of EBV and high-risk HPVs in cervical tissues is more frequent in patients with high-grade squamous intraepithelial lesions in comparison with low-grade lesions (19). Thus, the presence of EBV in high-grade cervical lesions and cancer could suggest a possible cooperation between EBV and HPV in human cervical carcinogenesis; however, there are no studies regarding the co-presence of EBV and HPVs in the Middle East region.

Therefore, in this study, we evaluated the co-presence of these viruses and their association with Id-1 expression in cervical cancers in Syrian women. Our study pointed out that EBV and high-risk HPVs are co-present in 34% of our samples; more significantly, we noted that the co-incidence of these viruses is associated with poorly differentiated squamous cell carcinomas, which is accompanied with Id-1 overexpression.

## Materials and Methods

### EBV and HPV Detection

Formalin fixed paraffin embedded blocks of cervical cancer were obtained from 44 Syrian patients with an average age of 57.25 years. Paraffin-embedded cervical tumor tissues were obtained from the Department of Pathology, Faculty of Medicine at the University of Aleppo, Syria. The specimens and data used in this study were approved by the Ethics Committee of the Faculty of Medicine of Aleppo University, Syria. Five micrograms of purified genomic DNA (Qiagen GmbH, Hilden, Germany), from each sample, was analyzed for EBV and HPV by polymerase chain reaction (PCR) using specific primers for LMP1 and EBNA1 as well as E6/E7 of HPV types 16, 18, 31, 33, 35, 45, 51, 52, and 58, while, primers for GAPDH gene were used as an internal control (Tables 1 and 2). This analysis was performed as previously described by our group (9, 20).

**Table 1:** The specific primer sets for LMP1 and EBNA1 genes of Epstein–Barr virus used for polymerase chain reaction (PCR) amplification.

Genes	Primers
LMP1	5′-TTGGAGATTCTCTGGCGACT-3′
	5′-AGTCATCGTGGTGGTGTTCA-3′
EBNA1-297	5′-AAGGAGGGTGGTTTGGAAAG-3′
	5′-AGACAATGGACTCCCTTAGC-3′
EBNA1-207	5′-ATCGTGGTCAAGGAGGTTCC-3′
	5′-ACTCAATGGTGTAAGACGAC-3′
GAPDH	5′-GAAGGC-CATGCCAGTGAGCT-3′
	5′-CCGGGAAACTGTGGCGTGAT-3′

**Table 2:** Epstein–Barr virus (EBV) and high-risk HPVs detection in human cervical carcinomas.

Cervical cancer samples (*n* = 44)	HPV status^a^	EBV status^a^
Positive	42/44 (95%)	15/44 (34%)
Negative	2/44 (5%)	29/44 (66%)

*^a^Based on PCR and immunohistochemistry (IHC) assays*.

The co-incidence of these viruses was found in 15 (34%) samples out of 44 examined by PCR and IHC using specific primers for LMP1, EBNA1, and E6/E7 genes of EBV and high-risk HPVs types (16, 18, 31, 33, 35, 45, 51, 52, and 58) as well as monoclonal antibodies for LMP1 and E6, as described in the Section “Materials and Methods.”

### Tissue Microarray (TMA)

The TMA construction was achieved as illustrated previously by our group (21, 22). Briefly, cervical cancer samples were embedded into a virgin paraffin TMA block using a manual tissue arrayer (Beecher Instruments, Silver Spring, MD, USA). Each block was assembled without previous knowledge of linked clinical or pathological staging information. Two TMA cores of 1.0 mm in diameter were sampled from a cohort of 44 block tissue samples of Syrian patients diagnosed with cervical carcinomas. Afterward, 4 µm sections were cut and stained with hematoxylin and eosin on the initial slides to verify the histological diagnosis. Next, slides of the completed blocks were used for immunohistochemistry (IHC) analysis.

### Immunohistochemistry

Immunohistochemistry procedures examining the expression of LMP1, E6, and Id-1 were carried out using standard practices as follows. To analyze the protein expression patterns of LMP1, E6, and Id-1 in TMA slides, each one was deparaffinized in graded alcohol, rehydrated, and boiled (microwave) in 10 mM citrate buffer (pH 6.0) for antigen retrieval. Then, TMA slides were incubated for 35 min at 37°C with primary monoclonal and polyclonal antibodies for LMP1 of EBV and E6 of HPV as well as Id-1 (clone 1–4, clone C1P5, sc-488, from Dako and Calbiochem, Canada; as well as Santa Cruz Biotechnology, USA, respectively) using an automated immunostainer (Ventana Medical System, Tuscon, AZ, USA). The automated Ventana Medical System uses an indirect biotin–avidin system with a universal biotinylated immunoglobulin secondary antibody. Afterward, slides were counterstained with hematoxylin prior to mounting; staining procedures were completed according to the manufacturer’s recommendations. Negative controls were obtained by omitting specific primary antibody for LMP1 and E6 as well as specific blocking peptides from Santa Cruz Biotechnology and antibody for Id-1 protein. Following IHC, two independent observers examined all TMA slides. The tumors were considered positive for LMP1, E6, and Id-1 onco-proteins if cancer cells exhibited positivity ≥1%. In case of LMP1 protein expression (EBV), we also evaluated the presence of viral infection in tumor-infiltrating lymphocytes and stromal cells. All IHC assays were evaluated using the Olympus light microscope (BX53); the slides were evaluated under magnifications 2×, 4×, 10×, and 20×.

### Statistical Analysis

Statistical evaluations were done using IBM SPSS Statistics (version 22; SPSS Inc., Chicago, IL, USA) and R. Data were calculated as nonparametric files. We utilized χ^2^ test with Yates correction to assess the significance of the association between cancer aggressiveness, Id-1 expression, and the co-presence of EBV and high-risk HPVs. Analysis of variance (ANOVA) test was used to analyze the differences among the group means.

## Results

We have recently explored the presence of high-risk HPVs in a cohort of 44 cervical cancer samples from Syrian women. Our previous study revealed that 42 (95.45%) of the 44 samples are high-risk HPVs positive and all cases were infected with more than one HPV type. Moreover, these data revealed that the most prevalent high-risk HPV types are 33 (24+/44), 16 (21+/44), 18 (18+/44), 45 (17+/44), 52 (13+/44), 58 (11+/44), 35 (9+/44), 51 (7+/44), and 31 (5+/44) (9) [for methodology used for PCR assay, please refer to Ref. (6)]. Herein, we further investigated the co-presence of EBV and high-risk HPVs in our 44 samples by PCR and IHC analysis using specific primers for LMP1 and EBNA1 as well as E6/E7 genes of EBV and HPVs, respectively (Table 1; Figure S1 in Supplementary Material) and monoclonal antibodies for LMP1 and E6, as described in the Section “Materials and Methods.”

**Figure 1:**
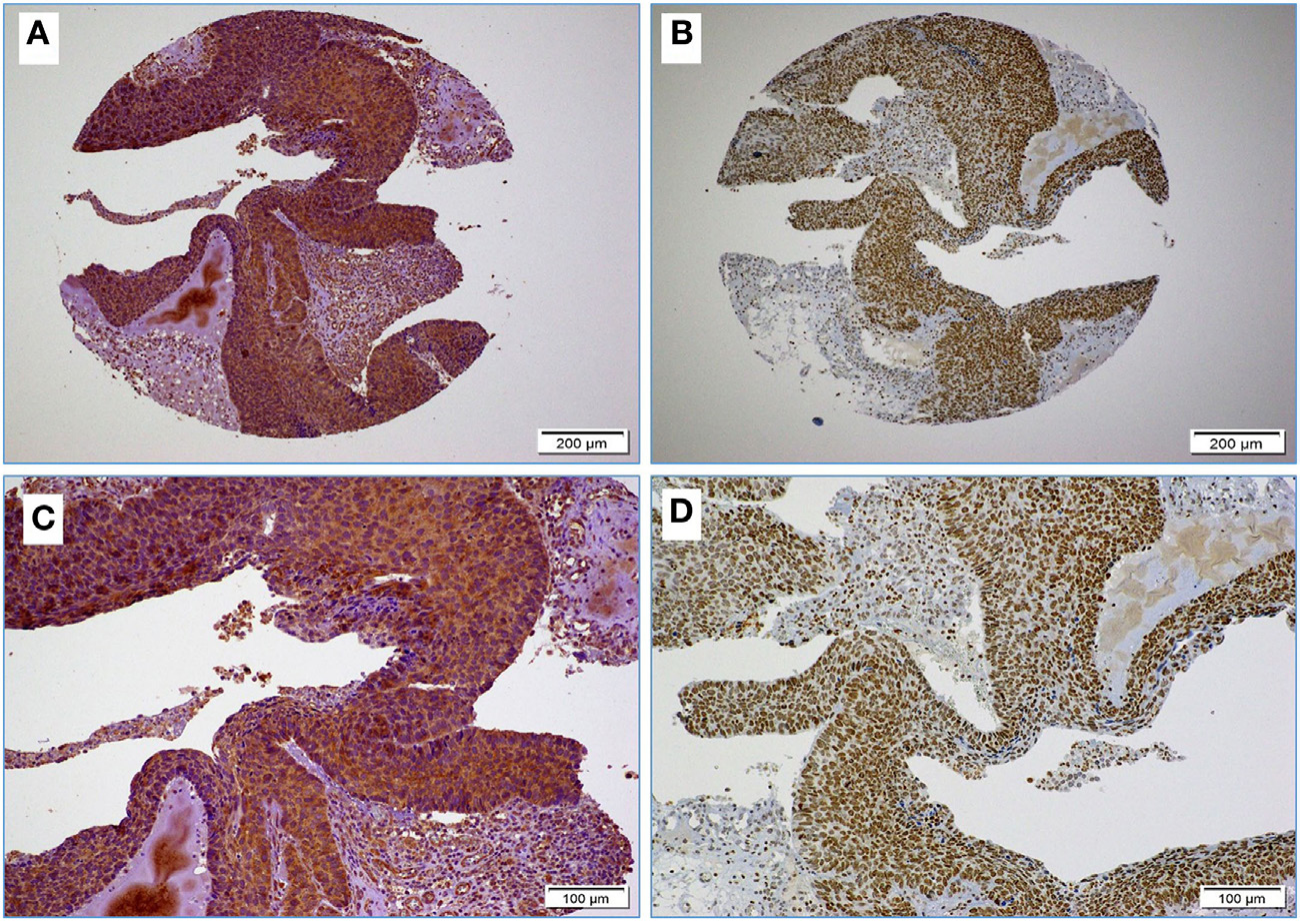
**(A,B)** Images reflect the diffused and strong cervical cancer cell positivity for high-risk HPV (E6 onco-protein) **(A)** and Epstein–Barr virus (EBV) (LMP1 protein) **(B)** (10× magnification); images **(C,D)** High-risk HPV and EBV positivity at higher magnification **(D)**; as shown, EBV positivity is clear in some stromal cells and tumor infiltrating lymphocytes (arrows) **(D)** (20× magnification).

We found that 15 (34%) of the 44 samples are positive (≥1% positive cancer cells) for both EBV and high-risk HPVs (Table 2; Figures 1A–D). None of the cases was exclusively EBV positive while two cases were both HPV and EBV negative. In addition, we found no statistically significant association between the various HPV types and EBV co-infection in cervical cancer samples (*p* > 0.05).

**Figure 2:**
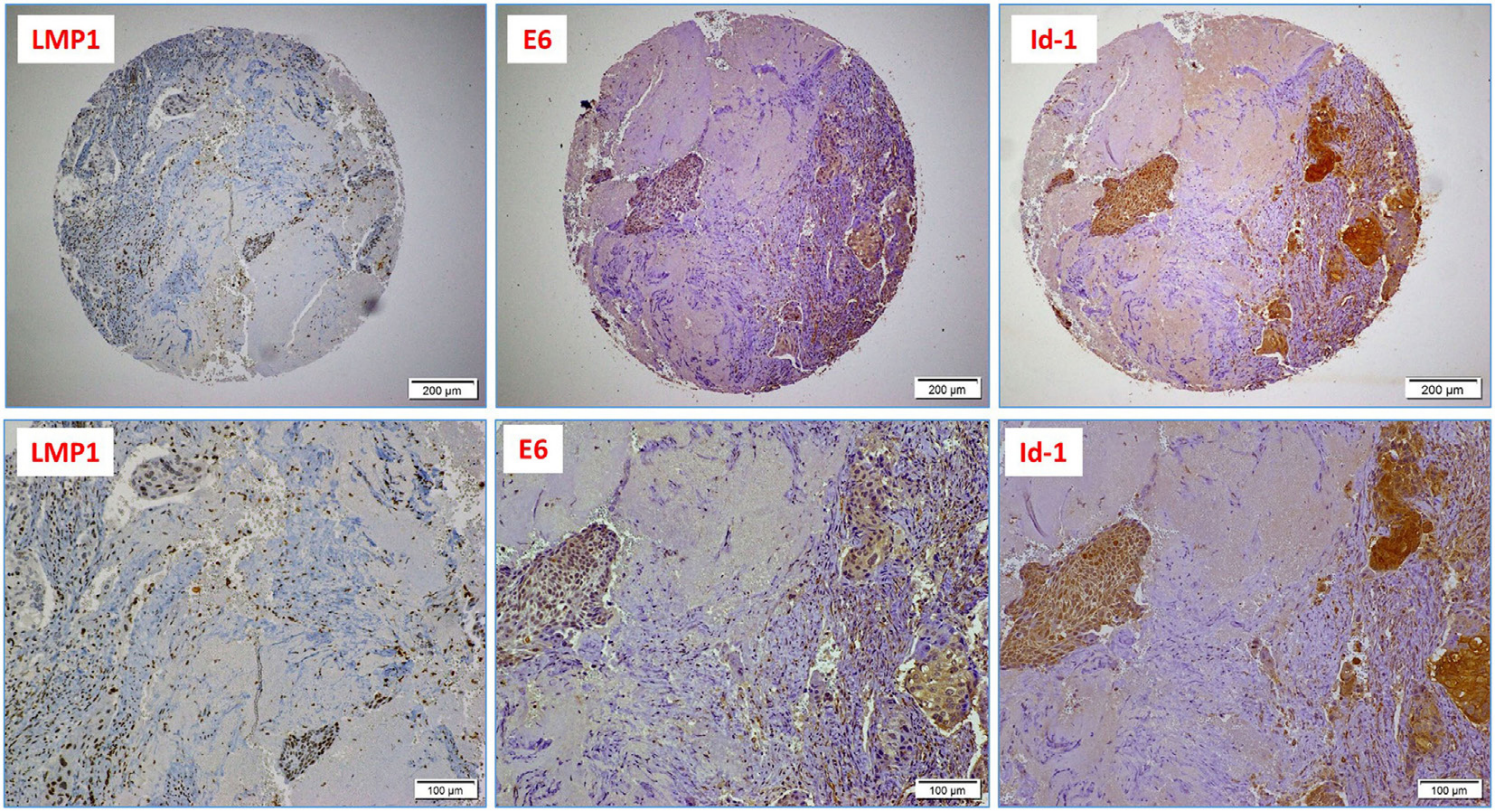
A case of poorly differentiated (high-grade, non-keratinizing) cervical carcinoma: upper images highlight the presence of Epstein–Barr virus (EBV) (LMP1 protein), high-risk HPV (E6 onco-protein), and a diffused Id-1 protein expression (10× magnification); lower images are respective high-power images (20× magnification); note the presence of EBV-positive tumor infiltrating lymphocytes (arrows).

Next, we assessed the association between the co-presence of these viruses and tumor phenotype in our samples using TMA methodology. Our data indicate that the co-expression of the LMP1 and E6 onco-proteins of EBV and high-risk HPVs, respectively, is associated with poorly differentiated squamous cell carcinoma form (Figure 2) in comparison with HPVs positive cases alone as well as negative cases for both, EBV and HPVs (*p* < 0.0001, respectively). On the other hand, we noted that the expression of LMP1 is located in cervical squamous cell carcinomas and frequently in stromal cells in addition to tumor infiltrating lymphocytes (Figure 1D); however, E6 of HPV, in general, is detected in cancer cells while the stromal and inflammatory cells (tumor infiltrating lymphocytes) are consistently negative (Figures 1D and 2).

Finally, we explored the association between the presence of EBV and HPVs with Id-1 overexpression in our Syrian samples by IHC. Using a 1% cutoff for positivity, Id-1 protein expression was observed in 41/44 cases (93%); diffuse and strong Id-1 expression (>50% cancer cells positive) was predominantly observed in high-grade (poorly differentiated) carcinomas (Figure 2). Moreover, we found that the co-expression of LMP1 and E6 (of EBV and HPV, respectively) is associated with diffuse and strong Id-1 overexpression in all invasive squamous cell carcinomas including high-grade carcinomas (*p* = 0.001) (Figure 2). In particular, the association between HPV (E6) and diffuse Id-1 (>50% cancer cells) was strong (*p* < 0.0001). ANOVA test for overall significance confirmed the observed differences between the subgroups (HPV+/EBV+ vs. HPV+ alone) and Id-1 status (*p* < 0.0001).

## Discussion

In this investigation, we explored, for the first time, the co-presence of EBV and high-risk HPVs in human cervical cancer and the role of this co-incidence with cancer phenotype in the conventional Middle East region. While, one study from North Africa pointed out that EBV and high-risk HPVs are co-present in 67.2% of cervical cancer cases in Algerian women (23). Herein, it is important to highlight that infection with, at least one high-risk HPV alone, is necessary but not sufficient to provoke cervical cancer initiation, additional oncovirus infection, and/or host genetic changes are required to drive neoplastic transformation and consequently lead to tumor formation (24, 25). In our investigation, we demonstrated that EBV is co-present with high-risk HPVs in 34% of cervical cancer cases in the Syrian population. Accordingly, a recent meta-analysis study of 25 investigations regarding the presence of EBV in human cervical cancer revealed that EBV is present in 43.63% of samples from cancer patients in comparison with 19% of samples from healthy people or patients with cervical intraepithelial neoplasia grade 1 (CIN) (27.34%) or CIN grade 2/3 (34.67%) (19). More significantly, co-infection with EBV and HPV is present in most of the cases, which display a similar phenotype of EBV infection (19); moreover, EBV infection is associated with differentiation (grade) of cervical epithelial cells (18). On the other hand, it has been pointed out that cervical carcinomas are four times more likely to occur among EBV-positive patients as compared with patients without EBV infection (19), which suggests a strong cooperation between EBV and HPVs in cervical carcinogenesis and possibly other human carcinomas (5). This concurs with our findings regarding the co-presence of EBV and high-risk HPVs and their association with cervical carcinomas in all positive cases, all of which are high-grade invasive cancers. Likewise, we have recently reported that EBV and high-risk HPVs are co-present in 32% of human breast cancer samples and their co-presence is associated with high-grade breast carcinomas and positive axillary lymph nodes (22).

On the other hand, it is important to highlight that EBV onco-proteins’ expression in cervical tissues is still controversial. Using *in situ* techniques for the detection of viral genomes or gene expressions, few investigations showed that EBV is present in cervical carcinoma cells (23, 26–28). However, others studies reported EBV localization in infiltrating lymphoid cells next to cervical carcinomas and concluded that EBV infection could not play a specific role in cervical carcinogenesis (29, 30). Interestingly, our study revealed that the expression of LMP1 protein is present in cervical squamous cell carcinomas and occasionally in the stroma as well as in tumor infiltrating lymphocytes; LMP1 is co-present with E6 onco-protein of high-risk HPVs in cervical carcinoma cells in most cases.

Concerning the association between the two oncoviruses (EBV and HPV) and Id-1 gene, which is overexpressed in several human carcinomas, it has been reported that LMP1 onco-protein of EBV upregulates the expression of Id-1 but not FoxO3a in human Hodgkin’s lymphoma cells (31). Likewise, in nasopharyngeal carcinoma, LMP1 induces an upregulation of Id-1 *via* FoxO3a inactivation (32). However, there are no studies regarding the EBV onco-proteins and Id-1 in human cervical cancer. In our present report, we demonstrate for the first time, the co-expression of LMP1 and E6 of EBV and high-risk HPVs, respectively, which is associated with Id-1 overexpression in human cervical cancer samples. However, herein, it is important to highlight that few investigations, including one from our lab, have pointed out that the presence of E6/E7 of high-risk HPVs is linked with Id-1 overexpression in human cervical cancer cells (9, 15, 33). More significantly, we have demonstrated that E6/E7 onco-proteins of HPV type 16 bind and active Id-1 promotor in human breast cancer cells; in parallel, we reported that Id-1 is the main regulator of cell invasion and metastasis induced by E6/E7 onco-proteins in these cancer cells (34). Accordingly, it is possible that EBV and high-risk HPV cooperate to upregulate the expression of Id-1 in human cervical cancer, which could enhance rapidly the progression of this cancer into invasive and metastatic form.

Nevertheless, further studies are necessary to clarify the role and pathogenesis of the co-presence of EBV and HPVs in human cervical carcinomas; especially since EBV and HPVs vaccines are presently under clinical trial and available, respectively (35–37). This is an important step, which could possibly limit cervical cancer initiation as well as its progression to a metastatic form, thereby decreasing cancer-related deaths especially in developing countries where cervical cancer is still the second major cause of death among women.

Finally, it is important to highlight that our investigation, in the Syrian population, is limited to a small number of cases located in a single region of Syria; therefore, it is essential to perform other studies of a larger number of cases from different regions in this country combined with several studies from the Middle East in general.

## Author Contributions

HA-T, SV, and AEA conceived the study. LG provided the samples and analyzed these data. HA-T, SV, TA, AY, GB, and AEA analyzed the data. All authors wrote and approved final version of the manuscript.

## Conflict of Interest Statement

The authors declare that the research was conducted in the absence of any commercial or financial relationships that could be construed as a potential conflict of interest.

## Supplementary Material

Figure S1 Representative polymerase chain reaction reactions for LMP1 of Epstein–Barr virus (EBV) in four cervical cancer samples. Chronic B leukemia cells were used as a positive control (PC); human normal cervical cells were utilized as negative control (NC).
