# Supplementary material for: Co-Incidence of Epstein–Barr Virus and High-Risk Human Papillomaviruses in Cervical Cancer of Syrian Women
Source: Front Oncol. 2018 Jul 2;8:250. doi: 10.3389/fonc.2018.00250 (PMC6043788; doi:10.3389/fonc.2018.00250)
Supplement: Figure S1 — Representative polymerase chain reaction reactions for LMP1 of Epstein–Barr virus (EBV) in four cervical cancer samples. Chronic B leukemia cells were used as a positive control (PC); human normal cervical cells were utilized as negative control (NC). [file presentation_1.pptx]

## Slide 1
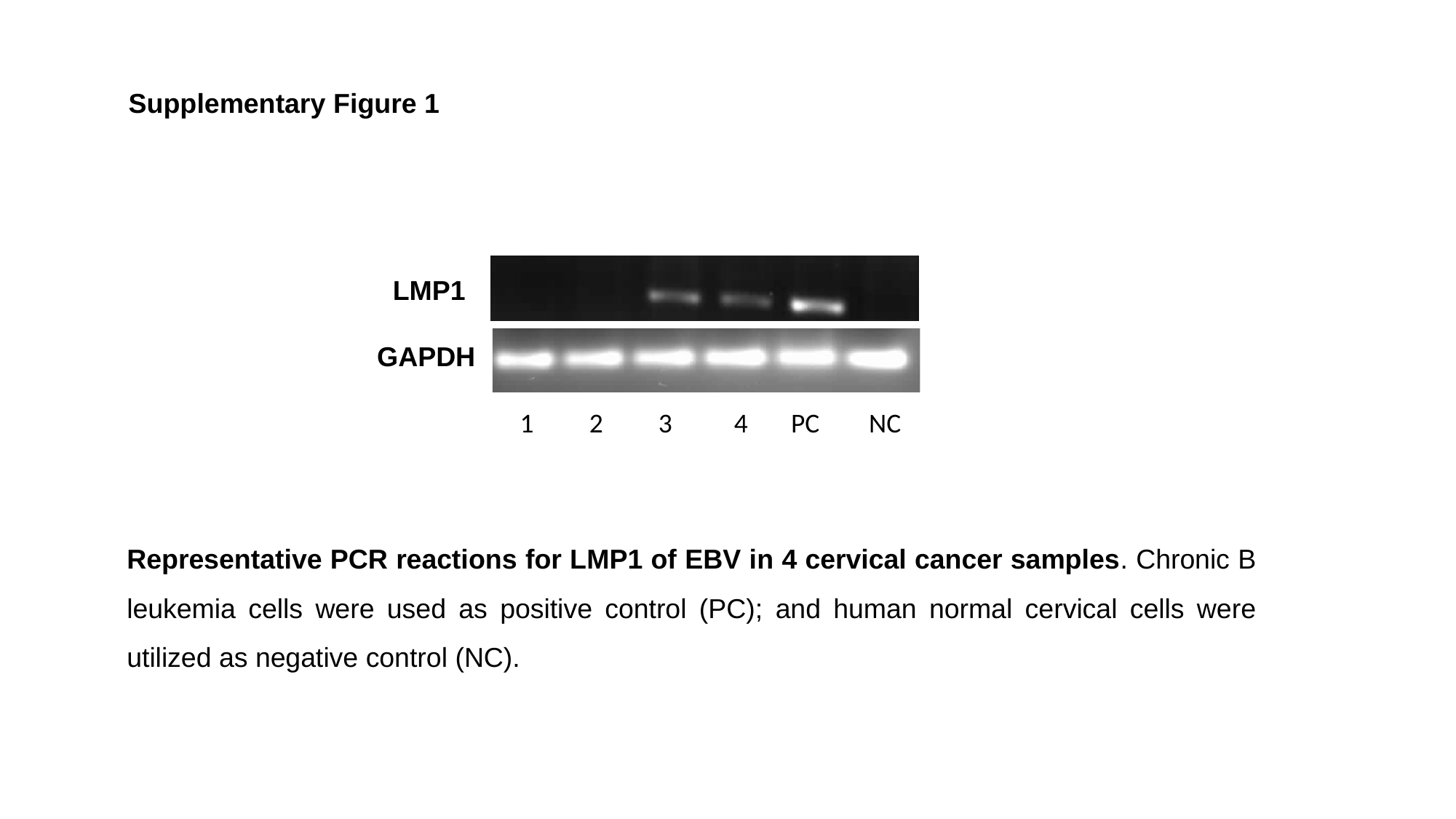

Supplementary Figure 1
LMP1
GAPDH
 1 2 3 4 PC NC
Representative PCR reactions for LMP1 of EBV in 4 cervical cancer samples. Chronic B leukemia cells were used as positive control (PC); and human normal cervical cells were utilized as negative control (NC).
